# Reply to: vitamin D supplementation and hemoglobin: dosing matters in prevention/treatment of anemia

**DOI:** 10.1186/s12937-021-00683-8

**Published:** 2021-04-08

**Authors:** Seyyed Mostafa Arabi, Golnaz Ranjbar, Leila Sadat Bahrami, Abdolreza Norouzy

**Affiliations:** Metabolic Syndrome Research Center, Mashhad University of Medical Sciences, Mashhad, 91179481564 Iran

Dear Editor:

We thank Professor Lena Napolitano for her insightful comments on our study. One of the concerns raised was regarding the correct interpretation of several words, such as the “length of intervention” mentioned in table 1; therefore, we have changed the duration of studies to intervention in order to include all of the items used in this manuscript. Furthermore, regarding two of the studies, namely by Smith et al., We used the publication years mentioned in the articles themselves, which are different from their citation years [1, 2].

**Fig. 1:**
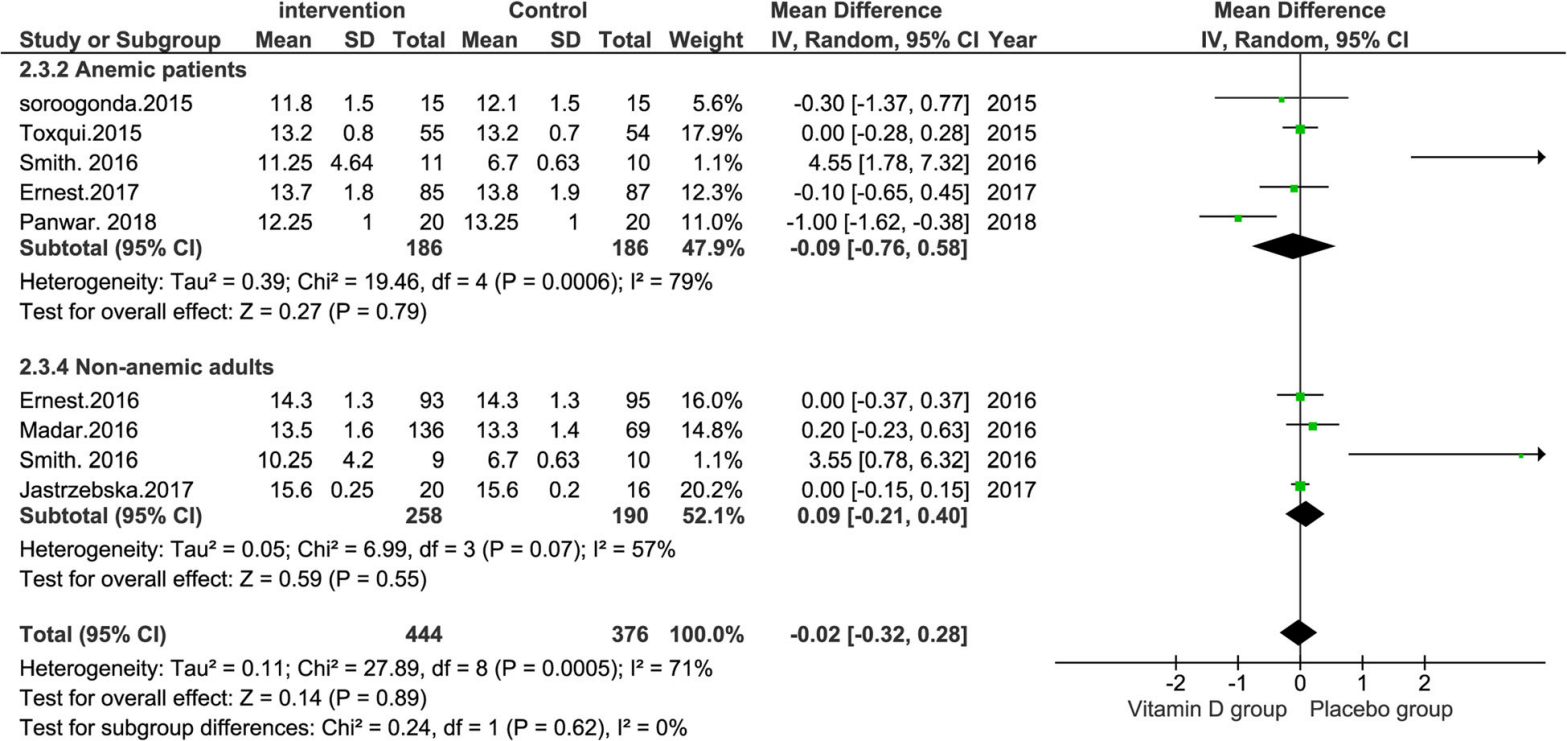
Forest plot showing results of a meta-analysis on the effects of vitamin D supplementation on hemoglobin in anemic and non-anemic adults. Data were reported as WMDs with 95% CIs

In our subgroup analysis, we divided the studies based on the general health conditions. However, per your recommendations, we also performed the analysis separately in anemic and non-anemic patients. As shown in Fig. 1, the effect of vitamin D was not significant in any of these groups.
